# Comprehensive Metabolomics and Network Pharmacology to Explore the Mechanism of 5-Hydroxymethyl Furfural in the Treatment of Blood Deficiency Syndrome

**DOI:** 10.3389/fphar.2021.811331

**Published:** 2022-03-04

**Authors:** Wensen Zhang, Na Cui, Fazhi Su, Yangyang Wang, Bingyou Yang, Yanping Sun, Wei Guan, Haixue Kuang, Qiuhong Wang

**Affiliations:** ^1^ Key Laboratory of Basic and Application Research of Beiyao (Heilongjiang University of Chinese Medicine), Ministry of Education, Harbin, China; ^2^ School of Traditional Chinese Medicine, Guangdong Pharmaceutical University, Guangdong, China

**Keywords:** 5-HMF, blood deficiency, network pharmacology, metabolomics, Radix Rehmanniae

## Abstract

Radix Rehmanniae (RR, from Radix Rehmanniae (Gaertn.) DC.) is a natural medicine used in traditional Chinese medicine (TCM) since ancient times for the treatment of blood disorders. RR is steamed to get Rehmanniae Radix Praeparata (RP), which has a tonic effect on blood; the content of 5-hydromethylfurfural (5-HMF) increases more than four times after steaming. Studies have shown that 5-HMF has positive pharmacological effects on cardiovascular and hematological disorders. This study aimed to explore and verify the impact of 5-HMF on rats with chemotherapy-induced blood deficiency syndrome (BDS). Rats were given cyclophosphamide (CP) and acetophenhydrazine (APH) to induce BDS, the coefficients of some organs (liver, spleen, and kidney) were measured, and a routine blood test examined the coefficients of several peripheral blood cells. Metabolomics and network pharmacology were combined to find important biomarkers, targets, and pathways. Western blot was used to detect the expression of CYP17A1 and HSD3B1 proteins in the spleen. All these findings suggested that the 5-HMF significantly increased the number of peripheral blood cells and reversed splenomegaly in rats. In addition, 5-HMF upregulated CYP17A1 and HSD3B1 protein expression in splenic tissues. Also, 5-HMF ameliorated chemotherapy-induced BDS in rats, and its therapeutic mechanism might depend on steroid hormone biosynthesis and other pathways. It acts on blood deficiency via multiple targets and pathways, which is unique to Chinese medicine.

## 1 Introduction

According to the theory of traditional Chinese medicine (TCM), blood deficiency is a common disease and a pathological state of blood dysfunction and organ malnutrition (Li et al., 2015). Blood deficiency syndrome (BDS) is mainly related to excessive blood loss (Shi et al., 2014), deficient spleen and stomach function, insufficient hematogenesis, and blood stasis (Yong et al., 2012). The main diagnostic index is the decrease in blood cells or hemoglobin content, similar to anemia (Wang et al., 2013). Modern medicine holds that patients with BDS often have clinical manifestations such as impaired hematopoietic function, decreased visceral function, malnutrition, and bone marrow suppression (Zhang et al., 2014). BDS can cause spleen enlargement (Zhang et al., 2020). After chemotherapy, most patients have a hemoglobin synthesis disorder, which causes BDS and severely interferes with blood system circulation (Jiang et al., 2013). However, anemia is defined only as a decrease in the concentration of hemoglobin in the body, which is usually related to a reduction in red blood cells (Li et al., 2017).During cancer treatment, the standard treatment for BDS is iron and vitamin B12 supplementation to promote the synthesis of erythropoietin, which can accelerate the recovery of the hematopoietic system (Brown 2020). However, the application of these regimens is limited because of their unstable efficacy, high cost, and side effects (Milano and Schneider 2007).

A main component of TCM is 5-HMF, among which the most representative herb is Rehmanniae Radix Praeparata (RP) (Won et al., 2014). Rehmanniae Radix (RR) is steamed to obtain RP, which is a plant of the Scrophulariaceae family. RP has been used to treat BDS for thousands of years. It is a commonly used TCM in Asia (Li et al., 2021). The Compendium of Materia Medica records that RP is used as a blood tonic (Guo et al., 2016), which regulates and fortifies blood and treats BDS (Zee-Cheng 1992). The main components of RR are monosaccharides, oligosaccharides (Kitagawa et al., 1995), iridoids (Xia et al., 2020), and glycosides (Xu et al., 2012). The decomposition products of these compounds constitute RP, of which 5-HMF is the most prominent (Won et al., 2014). During the processing of RR, stachyose is reduced to hexose, and these components finally form 5-HMF, which increases the content of 5-HMF in RP after steaming by more than four times compared with RR (Zhu et al., 2007). Therefore, 5-HMF has become the main active component of RP and is used as a marker compound for quality control (Li et al., 2005; Korean Food and Drug Administration, 2008). This transformation of active ingredients in raw materials and processed products leads to differences in their efficacy. For example, RP is better at supplementing blood (Xia et al., 2020). According to current research results, 5-HMF has positive pharmacological effects on cardiovascular and blood diseases. For example, 5-HMF can increase hemoglobin’s oxygen affinity to support cardiac function during severe hypoxia (Lucas et al., 2019). It also has sound biological effects, such as antioxidant activity (Li et al., 2011) and strengthening the resistance of sickle red blood cells to injury caused by hypoxia (Qiang et al., 2021). Therefore, we speculate that 5-HMF can be used as a potential therapeutic drug for BDS. However, the treatment of BDS by 5-HMF has not been documented, and its therapeutic mechanism is unclear.

Network pharmacology and metabolomics are practical tools for elucidating the underlying mechanisms of TCM (Pan et al., 2020). First, a blood deficiency rat model induced by chemotherapy was used to evaluate the effect of 5-HMF on BDS. Second, a network pharmacology method was established to explore the mechanism of 5-HMF in treating BDS from the perspective of targets and pathways. At the same time, plasma metabolomics revealed the synergistic metabolic mechanism of metabolites and metabolic pathways. Finally, Western blot validated the metabolic pathway. This study was the first to use a combination of network pharmacology and plasma metabolomics to determine the mechanism of 5-HMF in the treatment of BDS. Hopefully, these results will provide a theoretical basis for elucidating the mechanism of 5-HMF in BDS treatment.

## 2 Materials and Methods

### 2.1 Animals and Ethic Statement

We used 40 healthy male SD rats weighing 220–260 g, batch number SCXK (Liao) 2020–0001, from Liaoning Changsheng Biotechnology Co., Ltd. The animal experiment was approved by the Ethics Committee of Heilongjiang University of Traditional Chinese Medicine (approval number 2019121101). Animals were kept in a room at 25 C and 40–60% humidity. Rats were randomly divided into five groups with 8 rats in each group: control group, model group, 5-HMF-L group, 5-HMF-M group, and 5-HMF-H group.

### 2.2 Chemicals and Reagents

The 5-HMF was purchased from Aladdin (batch number H2020020, >95% (Gas chromatography), containing 3–5% water as a stabilizer). N-acetophenazine was purchased from Aladdin (batch number L1909182), and cyclophosphamide (CP) was purchased from MACKLIN (batch number C11147187).

### 2.3 Establishment of the Blood Deficiency Syndrome Model

The establishment of the BDS animal model was consistent with previous studies (Li et al., 2015). APH (20 mg/kg, 10 mg/kg) was injected subcutaneously on the first and fourth days. On the 4th day, 2 h after subcutaneous injection of APH, CP (20 mg/kg) was injected intraperitoneally. CP was injected for 4 consecutive days after that. On the 7th day, the indexes of red blood cells (RBC), white blood cells (WBC), hemoglobin (HGB), and hematocrit (HCT) in peripheral blood were measured. The control group was injected with the same volume of saline in the same way. From the first day, the treatment group was given 5-HMF (5.19 mg/kg, 2.595 mg/kg, 1.2975 mg/kg) by gavage (Duan 2021), respectively, and the control group and model group were given the same volume of saline by gavage for 15 days.

### 2.4 Routine Blood Test

After the last administration, blood was taken from the abdominal aorta and collected in a sterile vacuum blood collection tube containing ethylenediaminetetraacetic acid (EDTA). The blood was analyzed by the HEMAVET 950 automatic hematology analyzer (Drew Scientific Group, Dallas, Texas, United States) to quantify RBC, WBC, HGB, and HCT.

### 2.5 Network Analysis

#### 2.5.1 Target Prediction

The targets of 5-HMF were predicted in the following ways: Traditional Chinese Medicine Systems Pharmacology Database and Analysis Platform (TCMSP) (Ru et al., 2014), Swiss Target Prediction (Gfeller et al., 2014), Targetnet (Yao et al., 2016), PharmMapper (Wang et al., 2017). We used GeneCards (Stelzer et al., 2016), Online Mendelian Inheritance in Man (OMIM) (Amberger et al., 2015), Drugbank (Wishart et al., 2018) to identify targets related to BDS. In the OMIM database, we opened the Gene Map and entered “blood deficiency syndrome” to obtain 532 essential genes. We entered “blood deficiency syndrome” in the GeneCard database, selected genes with a relevance score >20, and obtained a total of 1,542 critical genes. We entered “anemia” in the Drugbank database and got a total of 11 essential genes and then removed the duplicated genes.

#### 2.5.2 Kyoto Encyclopedia of Genes and Genomes and Gene Ontology Enrichment Analysis

In analyzing the common targets of the 5-HMF action target and BDS-related targets, the server Metascape (Zhou et al., 2019) was used for Kyoto Encyclopedia of Genes and Genomes (KEGG) and Gene Ontology (GO) enrichment analysis.

#### 2.5.3 Network Construction

Two visualization networks were constructed: 1) Target point network, a network composed of 5-HMF, BDS, and common targets; 2) Targetpath network, in which the targets and their pathways were used to generate spatiotemporal networks. All networks were constructed by Cytoscape 3.8.0 (Liu et al., 2020).

### 2.6 LC-MS Metabolomics Analysis

#### 2.6.1 Sample Preparation

After the final administration, the blood of rats was collected from the abdominal aorta, left to stand for 30 min at 4 C, centrifuged at 3,500 rpm/min for 15 min. Then the plasma supernatant sample was mixed 1:3 with acetonitrile, vortexed for 30 s, and left to stand for 30 min at −20°C. The blood was centrifuged at 13,500 rpm/min for 15 min at 4 C, 300 μL of supernatant was evaporated, and 150 μL of 50% acetonitrile was added to redissolve the sample. After centrifugation, the sample was injected into the injection cup for testing (Gong et al., 2019).

#### 2.6.2 UPLC-QTOF/MS Analysis

The G2-Si (Waters Q-TOF SYNAPT from Manchester Waters Company) has a continuous mode and is equipped with an electrospray ion source. The plasma samples were chromatographed on an Acquity UPLC HSS T3 column with a gradient elution program at the flow rate of 0.2 μL/min. The mobile phase was comprised of water and 0.1% formic acid (phase A) and acetonitrile and 0.1% formic acid (phase B). The elution gradient of phase B was as follows: 2% (0–0.5 min), 2% up to 40% (0.5–9 min), 40% up to 98% (9–16 min), then maintained at 98% for 1 min. Optimized parameters for the mass spectrometer were as follows: ion spray voltage, 5 or −4 kV (for positive and negative mode, respectively); declustering potential, 60 V; curtain gas, 25 psi; nebulizer gas of 40 psi; interface heater temperature, 650 C; scan range (m/z), 50–1,200. The stability of the analysis was continuously monitored by analyzing QC samples at intervals of every 10 samples.

#### 2.6.3 Multivariate Data Analysis

The advanced Progressive QI platform (Waters, United States) was used to obtain potential and critical annotations from the original centroid data file obtained by SYNAPT G2-Si. Metabolic data completed by principal component analysis and dimensionality reduction had the most significant impact on health and disease. The potential differences in metabolites were identified from the orthogonal partial least squares discriminant analysis (OPLS-DA). The components that significantly impacted the grouping were screened out through objective numerical values. The *t*-test value was lower than 0.05.

#### 2.6.4 Biomarker Identification and Metabolic Pathway Analysis

LC-MS/MS data were imported into Ezinfo software (Waters Progressional QI) for principal component analysis and OPLS-DA data analysis. The variable projection importance value obtained by OPLS-DA was used to evaluate the impact of each metabolite difference on the discrimination and interpretation ability of each sample. The greater the VIP value, the more significant the contribution of metabolites to sample differentiation. It is generally believed that there are significant differences between variables with VIP >1. The potential biomarkers were the S-plot constructed by Ezinfo, and the V-plot was labeled.

### 2.7 Statistical Analysis

All data were expressed in terms of mean ± standard deviation. The two-tailed unpaired *t*-test was performed using SPSS 21.0 software (from SPSS Corporation, United States). A value of *p* < 0.05 was considered to show a significant difference. A value of *p* < 0.01 was considered to indicate a highly significant difference. The histogram was drawn by GraphPad Prism 7 software (GraphPad Software, United States).

#### 2.7.1 Pathway Analysis

The targets from network pharmacology and the metabolites from plasma metabolomics were jointly analyzed by MetaboAnalyst to select crucial metabolism pathways.

### 2.8 Western Blot Was Applied to Verify the Expression of Common Key Proteins in Metabolomics and Network Pharmacology

The bincinchoninic acid (BCA) method was used to determine the total protein of conventionally extracted splenocytes. The extract was separated by 10% SDS polyacrylamide gel electrophoresis and transferred to the PVDF membrane. It was blocked with 5% skim milk at room temperature for 1 h, and antibodies of CYP17A1, HSD3B1 (all 1:1,000), and GAPDH (all 1:2,000) were added. These were incubated overnight at 4 C and rinsed with tris buffered saline with Tween 20 (TBST) three times for 10 min each. The membrane was immersed in the horseradish peroxidase-labeled secondary antibody (1:1,000), diluted in 2% skimmed milk, incubated at room temperature for 1 h, and rinsed with TBST 3 times, each for 10 min. Image Quant LAS 500 imaging equipment acquired the signal using the ECL chemiluminescence method.

## 3 Results

### 3.1 Behavioral Analysis of Rats

Changes in the general behavior of rats could reflect the occurrence of and recovery from BDS in rats. After BDS induction, rats in the model group developed fatigue and lethargy, accompanied by weight loss, thinning hair, pale ears and tails, and loss of appetite. These symptoms were consistent with the description of BDS in Chinese medicine. In contrast, the control and BDS-induced rats treated with 5-HMF were relatively robust, showing thick and shiny hair, pink and moist nose and lips, round and pink tails, and stable body weight along with increased appetite.

### 3.2 Routine Blood Testing

**FIGURE 1:**
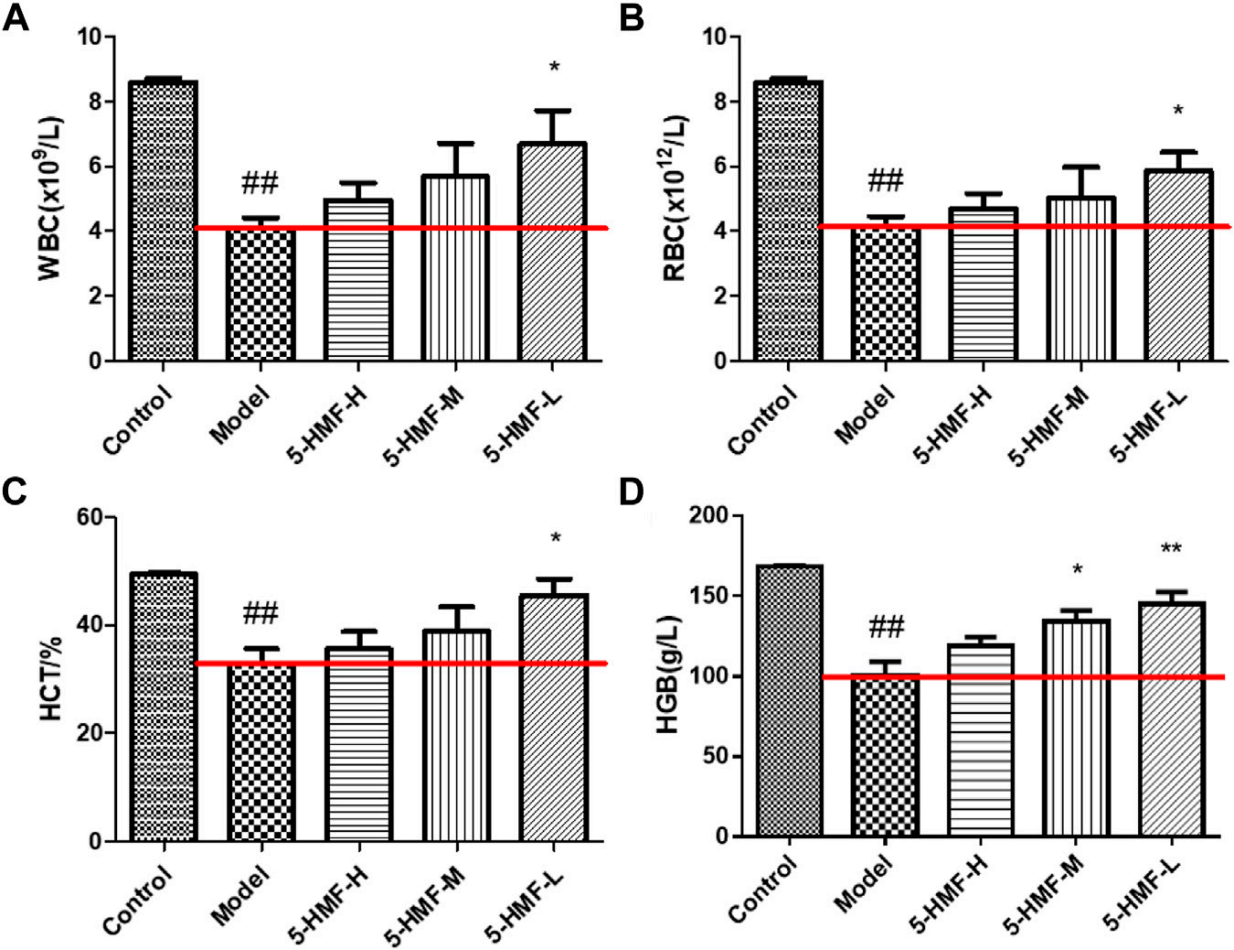
The blood parameters of rats in the control and model groups treated with saline and different doses of 5-HMF. **(A)** The level of WBC. **(B)** The level of RBC. **(C)** The level of HGB. **(D)** The level of HCT. Each value represents the mean SD (*n* = 8); ^*#*^ *p* < 0.05 and ^##^ *p* < 0.01, compared with the control group; *p < 0.05 and **p < 0.01, compared with the model group.

After 15 days of administration of normal saline and 5-HMF, the RBC, WBC, HGB, and HCT levels in the peripheral blood of the rats were measured (Figure 1). Compared with the control group, the RBC, WBC, HGB, and HCT levels in the model group were significantly reduced (*p* < 0.01), indicating that the blood deficiency model was successfully established. The levels of WBC, RBC, HGB, and HCT in the Model group were significantly increased after 5-HMF treatment (*p* < 0.05 or *p* < 0.01).

### 3.3 Organ Index

**FIGURE 2:**
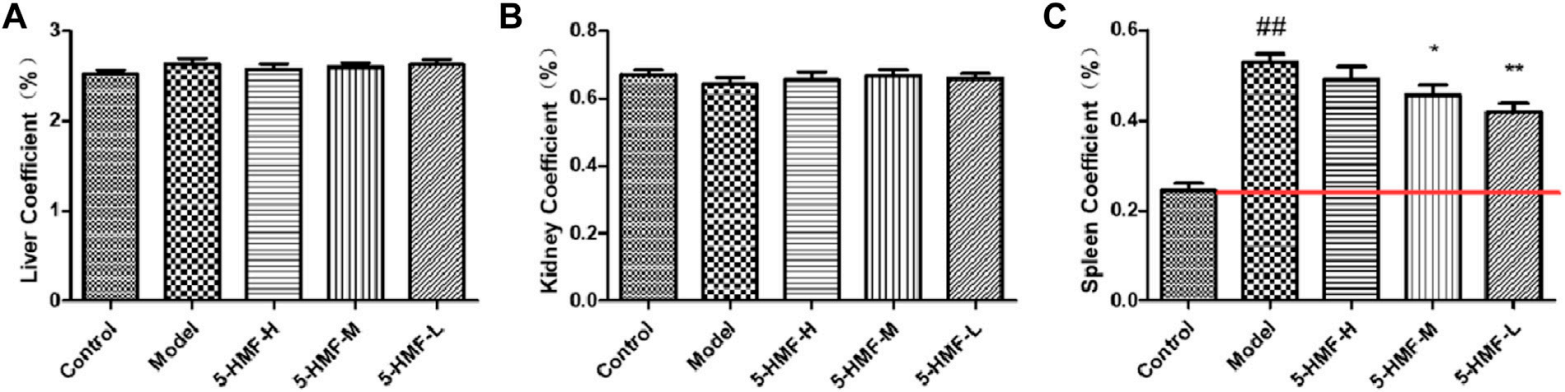
Organ index of mice in the control group and model group treated with saline (model) and different doses of 5-HMF. **(A)** Liver index. **(B)** Kidney index. **(C)** Spleen index. Each value represents the mean SD (*n* = 8); ^*##*^ *p* < 0.01, compared with the control group; *p < 0.05 and **p < 0.01, compared with the model group.

The results of the organ index are shown in Figure 2 Compared with the control group, the liver and kidney function in the model group were relatively unchanged, but the spleen was abnormally enlarged. Except for the spleen coefficient, the experimental group (model group, 5-HMF-H group, 5-HMF-M group, and 5-HMF-L group) and the control group had no significant differences in other organ coefficients. Compared with the control group, the spleen coefficient in the model group increased significantly (*p* < 0.01). Compared with the model group, the spleen coefficient in the 5-HMF-L group was significantly reduced (*p* < 0.01), the spleen coefficient in the 5-HMF-M group was decreased significantly (*p* < 0.05), and the spleen coefficient in the 5-HMF-H group was not very different. The results showed that 5-HMF had a dose-dependent reversal effect on spleen enlargement.

### 3.4 Results of Network Pharmacology Analysis

#### 3.4.1 5-Hydromethylfurfural and Blood Deficiency Syndrome Common Target Interaction

**FIGURE 3:**
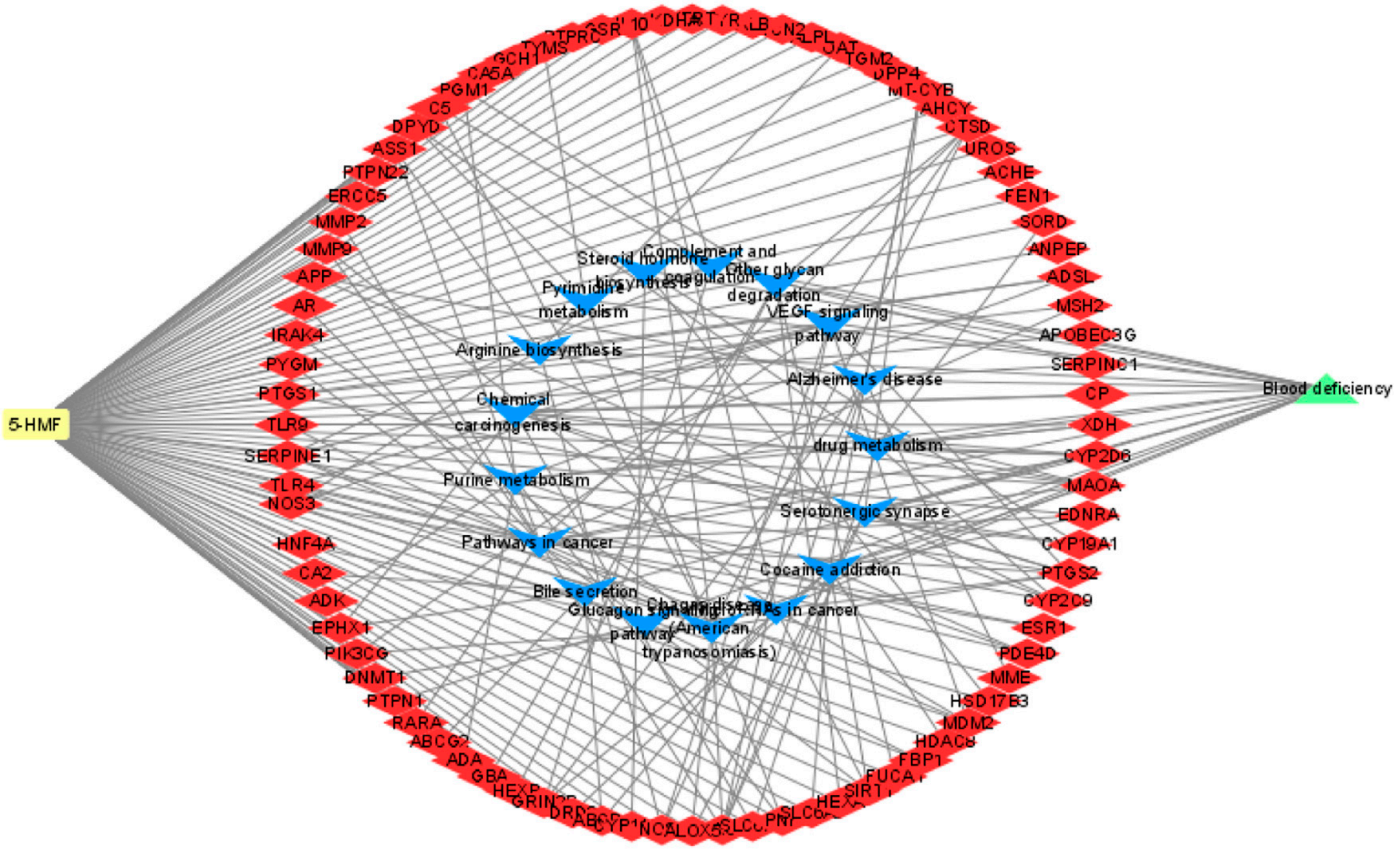
The blue triangle node represents the common pathway of 5-HMF and BDS, while the red quadrilateral node represents the common target of 5-HMF and BDS.

**FIGURE 4:**
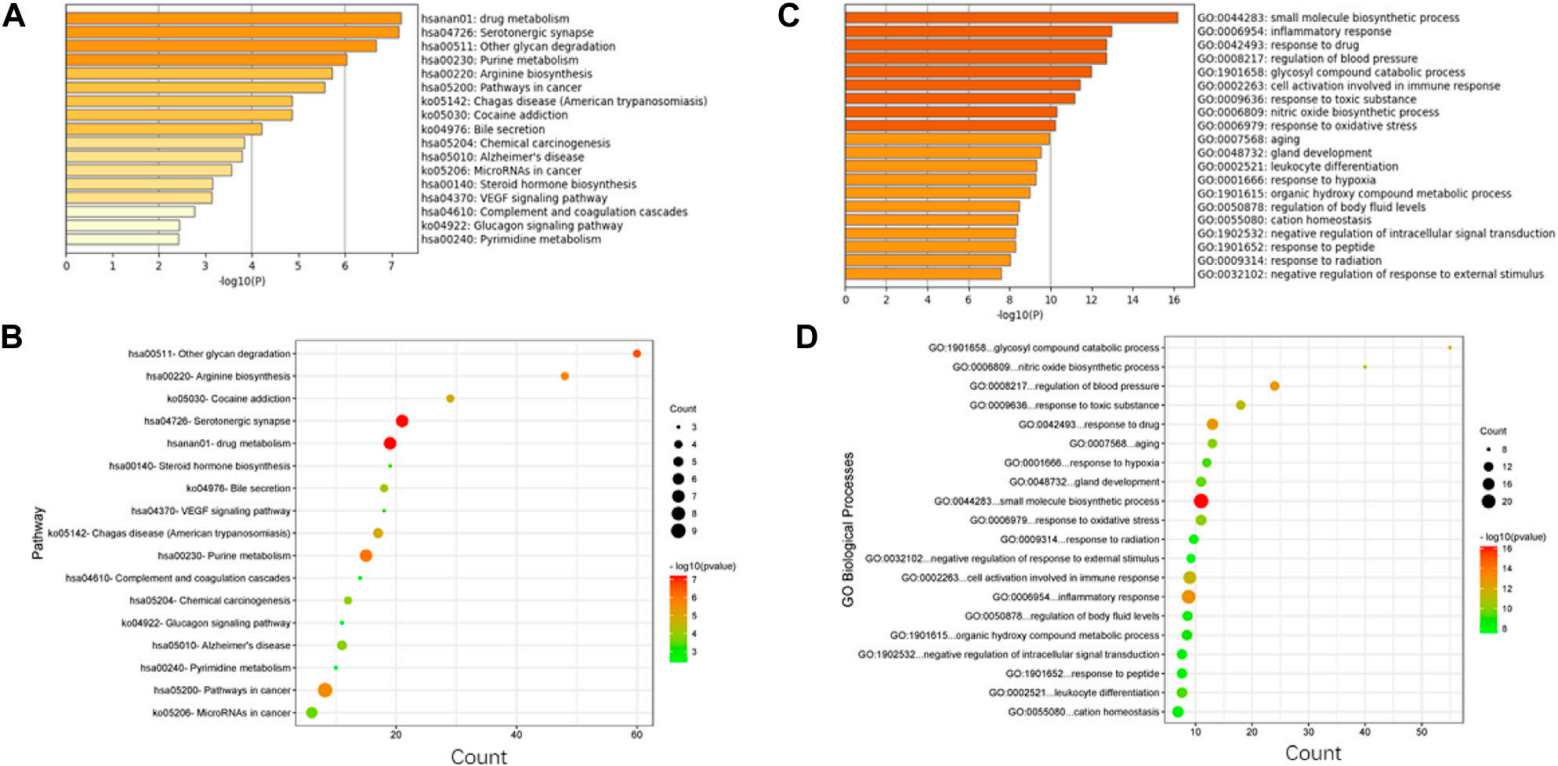
**(A)** 5-HMF and BDS common target KEGG enrichment analysis; **(B)** 5-HMF and BDS common target KEGG enrichment analysis bubble chart; **(C)** 5-HMF and BDS common target GO enrichment analysis of dots; **(D)** Bubble chart of GO enrichment analysis of the common target of 5-HMF and BDS.

In this study, four online websites were used to predict 5-HMF targets, and three were used to predict BDS disease targets. Finally, it was determined that 5-HMF had 494 targets, BDS had a total of 1887 targets, and a total of 84 common targets. To further explore the mechanism of 5-HMF treatment of BDS, KEGG enrichment analysis was performed on 84 common targets, and 14 metabolic pathways were obtained (Figure 3), and the common targets and pathways are illustrated in a network diagram (Figure 4). The results show that the 5-HMF treatment of BDS has the characteristics of multiple pathways and multiple targets.

### 3.5 Results of Metabolomics Analysis

#### 3.5.1 Multivariate Data Analysis

**FIGURE 5:**
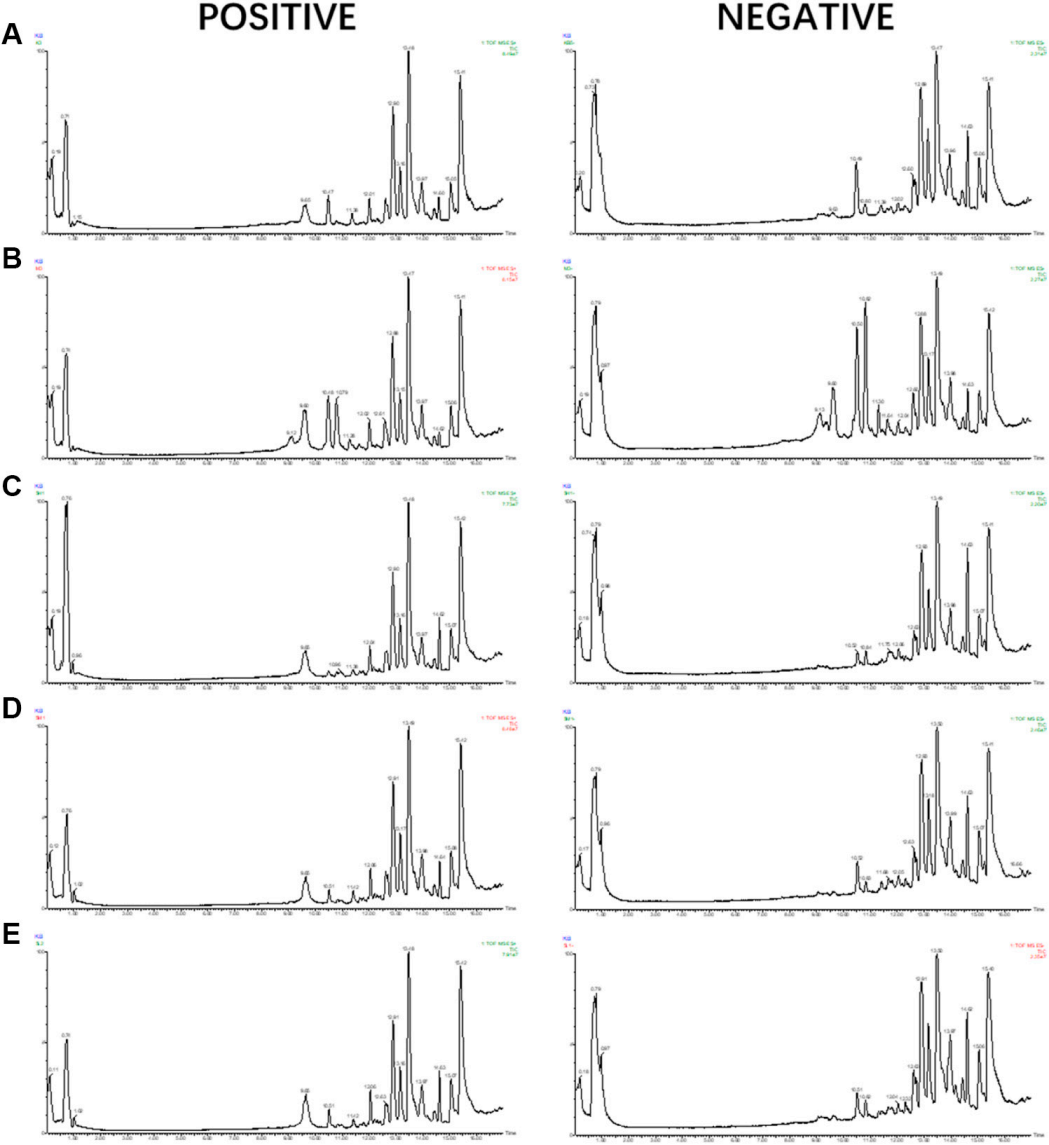
UPLC-MS/MS TIC diagram: **(A)** Control group, **(B)** Model group **(C)** 5-HMF-H group, **(D)** 5-HMF-M group, **(E)** 5-HMF- L group.

**FIGURE 6:**
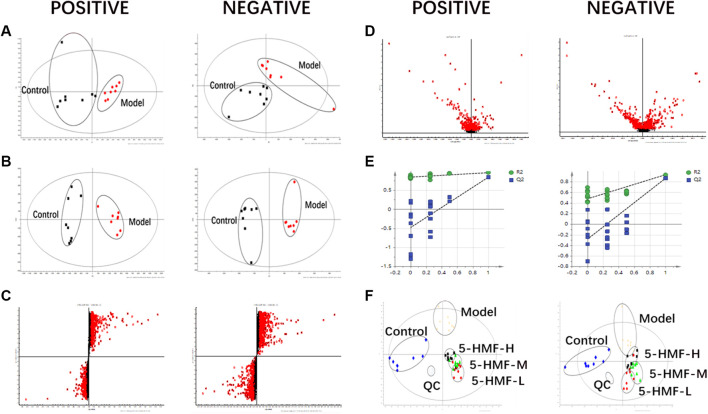
Multivariate data of UPLC-MS/MS: **(A)** Principal Component Analysis Score Chart, **(B)** OPLS-DA Analysis Score Chart **(C)** OPLS-DA S-plot Chart, **(D)** OPLS-DA V-plot Chart **(E)** Partial Least Squares-Data Analysis Model Validation Diagram, **(F)** PLS-DA Analysis Score Diagram. Plasma samples were collected from the different groups.

The samples were analyzed with the UPLC/Q-TOF metabolic spectrum. No abnormalities were found in the total ion chromatogram, and the retention time of chromatographic peaks and their ionic strength were observed (Figure 5) Masslynx and Progenesis QI software were used to analyze many raw files from G2-Si total quantification for data preprocessing, such as peak picking, denoising, and normalization, to generate a bioinformatics matrix. Then we exported the data set to Simca 14.1 software for chemometric analysis. The principal component analysis score chart showed that the model group was separated from the control group (Figure 6A). A significant separation between the model group and the control group was observed in the OPLS-DA chart (Figure 6B). The S-plot of OPLS-DA showed multiple metabolites (Figure 6C). The V-plot diagram of OPLS-DA showed a remarkable diversity of various metabolites (Figure 6D). The replacement test was used to verify the OPLS-DA mode (Figure 6E). Figure 6F shows that all experimental groups were separate in the liquid chromatography-mass spectrometry metabolic map. Among them, the metabolic profiles of the 5-HMF-H group, 5-HMF-M group, and 5-HMF-L group were closer to the control group than the model group, suggesting that the model’s metabolic disorder that was induced was reversed after drug treatment, and the 5-HMF-L group had a better separation effect. The four points in the QC group were in clustered, which proved that the machine was stable.

#### 3.5.2 Identification of Endogenous Metabolites

The difference metabolites between the control group and the model group were screened according to the VIP value (VIP >1.0) and *t*-test (*p* < 0.05). As a result, a total of 65 different metabolites were screened (Table 1). Compared with the control group, 35 different metabolites (epitestosterone, etc.) were significantly increased, and 30 different metabolites (17a-hydroxypregnenolone, etc.) significantly reduced. The 5-HMF-H group could regulate 48 metabolites (p-xanthine, etc.). The 5-HMF-M group could regulate 50 kinds of metabolites (5-KETE, etc.). The 5-HMF-L group could regulate 57 metabolites (5-KETE, etc.). The 5-HMF-L group had significantly more metabolic differences than the other two groups.

**TABLE 1:** Detection of BDS-related Metabolites in Plasma by LC-MS. Trend 1 is Control group compared with Model group; Trend 2 is Model group compared with 5-HML-L group.

NO.	Metabolites	TR (min)	m/z	Formula	VIP	*p* Value	Fold change	Trend 1	Trend 2	HMDB ID	Scan mode
1	PC(20:5 (5Z,8Z,11Z,14Z,17Z)/P-16:0)	20.98	764.5597	C_44_H_78_NO_7_P	1.34	4.60E-02	1.14	↑	—	8,521	+
2	PC(18:2 (9Z,12Z)/P-16:0)	20.91	764.5466	C_42_H_80_NO_7_P	1.51	3.11E-02	1.10	—	↑	8,159	+
3	Trihexosylceramide (d18:1/12:0)	20.71	985.6484	C_48_H_89_NO_18_	1.39	4.09E-02	1.21	—	↓	4,877	+
4	Trihexosylceramide (d18:1/16:0)	20.70	1,041.7068	C_52_H_97_NO_18_	4.17	6.76E-05	1.57	—	↓	4,879	+
5	LysoPC(22:2 (13Z,16Z))	20.68	593.4293	C_30_H_58_NO_7_P	7.26	5.55E-08	1.47	—	↓	10400	+
6	DG (16:0/20:0/0:0)	20.67	647.5559	C_39_H_76_O_5_	1.90	1.26E-02	1.12	—	↓	7,107	+
7	DG (16:0/18:0/0:0)	20.65	596.9646	C_37_H_72_O_5_	2.02	9.58E-03	1.31	↑	↓	7,100	+
8	CE (18:3 (9Z,12Z,15Z))	20.64	669.5554	C_45_H_74_O_2_	7.65	2.23E-08	1.72	—	↓	10370	+
9	2,5-Furandicarboxylic acid	20.59	201.0376	C_6_H_4_O_5_	6.53	2.93E-07	1.30	—	↑	4,812	-
10	Phytofluene	20.39	542.9203	C_40_H_62_	3.83	1.46E-04	3.22	↑	—	2871	+
11	Oleamide	20.27	282.2829	C_18_H_35_NO	2.39	4.10E-03	1.64	↑	—	2117	+
12	PC(20:0/18:4 (6Z,9Z,12Z,15Z))	20.18	810.1348	C_46_H_84_NO_8_P	2.18	6.63E-03	1.60	↓	—	8,273	+
13	PC(20:4 (8Z,11Z,14Z,17Z)/18:0)	20.12	810.1348	C_46_H_84_NO_8_P	3.93	1.16E-04	2.56	↓	—	8,464	+
14	PC(18:2 (9Z,12Z)/P-18:1 (11Z))	20.11	768.0981	C_44_H_82_NO_7_P	1.80	1.58E-02	1.41	↓	—	8,161	+
15	PC(16:1 (9Z)/22:2 (13Z,16Z))	20.07	810.5913	C_46_H_86_NO_8_P	2.95	1.12E-03	1.57	—	↓	8,020	-
16	PC(18:0/P-18:1 (11Z))	20.05	770.5976	C_44_H_86_NO_7_P	2.91	1.22E-03	1.44	—	↑	8,062	-
17	PC(16:0/P-18:1 (11Z))	20.02	742.5653	C_42_H_82_NO_7_P	2.05	8.90E-03	1.57	—	↓	7,996	-
18	LysoPC(22:1 (13Z))	19.95	577.7737	C_30_H_60_NO_7_P	1.69	2.05E-02	1.14	↓	-	10399	+
19	PC(18:0/14:0)	19.94	734.5788	C_40_H_80_NO_8_P	4.23	5.88E-05	1.48	—	↓	8,031	+
20	Arachidonic acid	19.94	304.4669	C_20_H_32_O_2_	2.93	1.18E-03	1.99	↑	—	1,043	-
21	PI(18:2 (9Z,12Z)/16:0)	19.94	852.5681	C_43_H_79_O_13_P	2.95	1.11E-03	1.22	—	↓	9846	+
22	PC(20:1 (11Z)/P-16:0)	19.94	794.6085	C_44_H_86_NO_7_P	8.07	8.47E-09	1.44	—	↑	8,324	+
23	PC(18:3 (9Z,12Z,15Z)/P-18:0)	19.94	768.5950	C_44_H_82_NO_7_P	2.40	3.98E-03	1.40	—	↑	8,226	+
24	PC(20:4 (8Z,11Z,14Z,17Z)/16:0)	19.93	782.0817	C_44_H_80_NO_8_P	9.10	7.91E-10	7.06	↑	—	8,462	+
25	PI(22:4 (7Z,10Z,13Z,16Z)/16:0)	19.93	887.5759	C_47_H_83_O_13_P	3.77	1.70E-04	1.13	—	↓	9914	+
26	PC(20:2 (11Z,14Z)/14:0)	18.77	758.0603	C_42_H_80_NO_8_P	1.85	1.40E-02	2.92	↑	—	8,328	+
27	PC(18:2 (9Z,12Z)/18:1 (9Z))	18.71	784.5924	C_44_H_82_NO_8_P	2.63	2.34E-03	1.26	—	↓	8,137	+
28	PI(16:0/20:0)	18.71	911.5975	C_45_H_87_O_13_P	4.67	2.16E-05	3.18	—	↑	9785	—
29	PC(18:4 (6Z,9Z,12Z,15Z)/18:1 (9Z))	18.71	780.0658	C_44_H_78_NO_8_P	7.19	6.53E-08	6.71	↑	—	8,236	+
30	Trihexosylceramide (d18:1/24:0)	18.71	1,158.7722	C_60_H_113_NO_18_	4.80	1.58E-05	1.26	—	↓	4,886	+
31	DG (16:1 (9Z)/18:2 (9Z,12Z)/0:0)	18.70	591.5034	C_37_H_66_O_5_	15.11	7.77E-16	3.03	—	↓	7,132	+
32	DG (16:0/16:0/0:0)	18.69	568.9114	C_35_H_68_O_5_	1.60	2.50E-02	1.15	↑	↓	7,098	+
33	CE (22:5 (7Z,10Z,13Z,16Z,19Z))	18.69	721.5887	C_49_H_78_O_2_	12.48	3.31E-13	2.25	—	↓	10375	+
34	D-Erythrose 4-phosphate	18.69	245.0038	C_4_H_9_O_7_P	5.49	3.20E-06	1.28	—	↑	1,321	-
35	LysoPC(22:6 (4Z,7Z,10Z,13Z,16Z,19Z))	18.67	567.6943	C_30_H_50_NO_7_P	3.99	1.02E-04	4.47	↓	-	10404	+
36	Glucosylceramide (d18:1/12:0)	18.63	644.5063	C_36_H_69_NO_8_	5.40	3.99E-06	1.25	—	↓	4,969	+
37	S-Adenosylmethionine	17.87	399.4450	C_15_H_23_N_6_O_5_S+	1.99	1.03E-02	1.86	↑	—	1,185	—
38	SM(d18:0/14:1 (9Z) (OH))	17.84	689.5316	C_37_H_73_N_2_O_7_P	2.41	3.92E-03	1.39	—	↓	13462	+
39	PC(16:1 (9Z)/P-16:0)	17.83	733.5758	C_40_H_78_NO_7_P	5.36	4.39E-06	1.45	-	↓	8,027	+
40	PC(20:3 (5Z,8Z,11Z)/P-18:1 (11Z))	17.82	816.6024	C_46_H_84_NO_7_P	5.09	8.06E-06	1.43	—	↑	8,392	+
41	DG (18:1 (9Z)/18:2 (9Z,12Z)/0:0)	17.82	618.9701	C_39_H_70_O_5_	2.48	3.33E-03	1.26	↑	—	7,219	+
42	Secoisolariciresinol	17.81	407.1669	C_20_H_26_O_6_	3.11	7.70E-04	1.32	—	↓	13692	—
43	PC(22:2 (13Z,16Z)/14:0)	17.81	786.1134	C_44_H_84_NO_8_P	2.20	6.34E-03	1.26	↓	—	8,590	+
44	17a-Hydroxypregnenolone	17.79	332.4770	C_21_H_32_O_3_	2.92	1.20E-03	1.61	↓	↑	363	+
45	PI(22:5 (7Z,10Z,13Z,16Z,19Z)/18:0)	17.68	913.1652	C_49_H_85_O_13_P	2.91	1.23E-03	2.77	↑	—	9919	—
46	DG (18:1 (9Z)/20:0/0:0)	17.61	668.6202	C_41_H_78_O_5_	6.38	4.20E-07	2.13	—	↓	7,223	+
47	PC(18:0/20:3 (8Z,11Z,14Z))	17.59	812.6229	C_46_H_86_NO_8_P	2.33	4.69E-03	1.41	—	↓	8,047	+
48	SM(d18:0/16:1 (9Z) (OH))	17.59	717.5618	C_39_H_77_N_2_O_7_P	1.98	1.05E-02	1.38	—	↓	13463	+
49	SM(d18:0/16:1 (9Z))	17.59	703.5811	C_39_H_79_N_2_O_6_P	1.40	3.97E-02	1.14	—	↓	13464	+
50	PC(20:3 (8Z,11Z,14Z)/14:0)	17.58	756.0444	C_42_H_78_NO_8_P	1.51	3.10E-02	1.39	↑	—	8,394	+
51	LysoPC(22:0)	17.57	579.7895	C_30_H_62_NO_7_P	2.26	5.51E-03	1.34	↓	—	10398	+
52	PI(16:0/18:0)	17.32	856.5954	C_43_H_83_O_13_P	5.57	2.71E-06	1.54	—	↑	9781	+
53	PC(22:5 (7Z,10Z,13Z,16Z,19Z)/16:0)	17.31	808.1189	C_46_H_82_NO_8_P	5.60	2.53E-06	3.07	↑	↓	8,692	+
54	Ceramide (d18:1/25:0)	17.29	664.1399	C_43_H_85_NO_3_	3.97	1.06E-04	11.72	↓	—	4,957	+
55	Glucosylceramide (d18:1/9Z-18:1)	17.19	726.0786	C_42_H_79_NO_8_	3.56	2.76E-04	1.47	↑	—	4,970	—
56	beta-Cryptoxanthin	16.94	552.8870	C_40_H_56_O	3.43	3.69E-04	1.90	↓	—	33844	+
57	9,10-Epoxyoctadecenoic acid	16.50	296.4449	C_18_H_32_O_3_	3.04	9.10E-04	1.27	↑	—	4,701	+
58	Adrenoyl ethanolamide	16.47	375.5878	C_24_H_41_NO_2_	2.10	7.90E-03	1.72	↑	—	13626	+
59	LysoPC(22:5 (4Z,7Z,10Z,13Z,16Z))	16.41	569.7101	C_30_H_52_NO_7_P	3.22	6.07E-04	1.56	↓	—	10402	+
60	Chenodeoxycholic acid	15.90	393.3044	C_24_H_40_O_4_	2.20	6.35E-03	1.09	—	↑	518	+
61	Cer(d18:1/14:0)	15.43	508.4804	C_32_H_63_NO_3_	1.55	2.85E-02	1.48	—	↓	11773	—
62	LysoPC(20:3 (5Z,8Z,11Z))	15.42	546.3631	C_28_H_52_NO_7_P	2.05	8.89E-03	1.11	—	↑	10393	+
63	PC(24:1 (15Z)/P-18:1 (11Z))	15.40	898.6832	C_50_H_96_NO_7_P	1.52	2.99E-02	6.92	—	↓	8,819	—
64	PC(22:2 (13Z,16Z)/P-18:1 (11Z))	15.36	868.6364	C_48_H_90_NO_7_P	1.59	2.58E-02	2.14	—	↓	8,621	—
65	Docosahexaenoic acid	14.85	328.4883	C_22_H_32_O_2_	3.07	8.49E-04	2.13	↑	—	2183	+
66	PC(14:0/18:3 (6Z,9Z,12Z))	14.63	745.5483	C_40_H_74_NO_8_P	8.01	9.74E-09	2.41	—	↓	7,875	+
67	Retinal	14.62	284.4357	C_20_H_28_O	1.42	3.83E-02	1.72	↑	—	1,358	+
68	Dihydrofolic acid	14.51	443.4133	C_19_H_21_N_7_O_6_	1.52	3.03E-02	2.22	↑	—	1,056	—
69	PC(20:5 (5Z,8Z,11Z,14Z,17Z)/P-18:1 (11Z))	14.46	834.5671	C_46_H_80_NO_7_P	4.13	7.48E-05	3.39	—	↑	8,523	—
70	N1,N12-Diacetylspermine	14.28	286.4136	C_14_H_30_N_4_O_2_	2.42	3.82E-03	3.41	↑	—	2172	—
71	LysoPC(22:4 (7Z,10Z,13Z,16Z))	14.25	571.7260	C_30_H_54_NO_7_P	3.14	7.20E-04	1.41	↓	↑	10401	—
72	LysoPC(20:2 (11Z,14Z))	14.20	547.7046	C_28_H_54_NO_7_P	2.72	1.89E-03	1.19	↓	—	10392	—
73	PC(20:4 (8Z,11Z,14Z,17Z)/P-18:1 (9Z))	14.06	792.1195	C_46_H_82_NO_7_P	3.61	2.47E-04	2.05	↑	—	8,491	—
74	PC(22:6 (4Z,7Z,10Z,13Z,16Z,19Z)/P-18:0)	14.05	818.1568	C_48_H_84_NO_7_P	4.43	3.73E-05	2.42	↑	—	8,752	—
75	PC(22:4 (7Z,10Z,13Z,16Z)/P-16:0)	13.98	794.1354	C_46_H_84_NO_7_P	2.53	2.98E-03	3.69	↑	—	8,652	—
76	all-trans-Retinoic acid	13.73	300.4420	C_20_H_28_O_2_	2.86	1.37E-03	3.46	↑	—	1852	+
77	PC(18:4 (6Z,9Z,12Z,15Z)/P-18:1 (11Z))	13.62	808.5438	C_44_H_78_NO_7_P	2.18	6.63E-03	2.28	—	↑	8,260	—
78	PS(18:0/18:1 (9Z))	13.60	788.5432	C_42_H_80_NO_10_P	2.71	1.93E-03	1.32	—	↓	10163	—
79	Ceramide (d18:1/12:0)	13.53	480.4446	C_30_H_59_NO_3_	1.76	1.75E-02	1.24	—	↓	4,947	—
80	Ubiquinone-1	13.43	250.2903	C_14_H_18_O_4_	1.82	1.52E-02	1.41	↓	↑	2012	+
81	L-Cystathionine	13.16	222.2620	C_7_H_14_N_2_O_4_S	3.21	6.19E-04	1.30	↓	↑	99	+
82	L-Palmitoylcarnitine	12.95	400.6230	C_23_H_45_NO_4_	4.43	3.70E-05	1.76	↑	—	222	+
83	Ganglioside GM3 (d18:1/16:0)	12.93	1,151.6951	C_57_H_104_N_2_O_21_	5.30	4.99E-06	1.24	—	↑	4,844	—
84	PS(18:0/22:6 (4Z,7Z,10Z,13Z,16Z,19Z))	12.69	836.0860	C_46_H_78_NO_10_P	5.28	5.30E-06	6.79	↓	—	10167	—
85	LysoPC(18:2 (9Z,12Z))	12.67	519.6515	C_26_H_50_NO_7_P	3.97	1.06E-04	1.26	↓	—	10386	—
86	5-KETE	12.41	318.4504	C_20_H_30_O_3_	5.22	6.06E-06	8.58	↓	—	10217	—
87	PS(18:0/20:4 (8Z,11Z,14Z,17Z))	12.40	812.0646	C_44_H_78_NO_10_P	3.45	3.54E-04	9.39	↓	—	10165	—
88	PC(18:3 (6Z,9Z,12Z)/P-18:1 (11Z))	12.40	810.5626	C_44_H_80_NO_7_P	3.30	4.96E-04	6.75	—	↑	8,194	—
89	13-cis-Retinoic acid	12.33	300.4351	C_20_H_28_O_2_	4.53	2.93E-05	7.08	↓	—	6219	+
90	LysoPC(16:1 (9Z)/0:0)	12.30	493.6142	C_24_H_48_NO_7_P	1.93	1.17E-02	1.45	↑	—	10383	+
91	2-Methoxyestrone	12.06	300.3921	C_19_H_24_O_3_	5.28	5.29E-06	5.28	↓	—	10	—
92	LysoPC(14:0/0:0)	11.80	467.5769	C_22_H_46_NO_7_P	1.56	2.73E-02	1.37	↑	—	10379	+
93	Testosterone glucuronide	11.74	464.5485	C_25_H_36_O_8_	3.81	1.56E-04	2.12	↑	—	3,193	-
94	Sphinganine	11.67	301.5078	C_18_H_39_NO_2_	2.85	1.43E-03	1.58	↑	—	269	+
95	Sphingosine 1-phosphate	11.65	379.4718	C_18_H_38_NO_5_P	4.85	1.42E-05	2.46	↑	—	277	+
96	15-Deoxy-d-12,14-PGJ2	11.51	316.4345	C_20_H_28_O_3_	5.39	4.09E-06	5.56	↓	↑	5079	—
97	Delta-12-Prostaglandin J2	11.34	334.4498	C_20_H_30_O_4_	2.79	1.64E-03	2.15	↓	—	4,238	—
98	Dehydroepiandrosterone	11.15	288.4244	C_19_H_28_O_2_	2.75	1.76E-03	2.16	↓	—	77	—
99	(9xi,10xi,12xi)-9,10-Dihydroxy-12-octadecenoic acid	10.93	314.4660	C_18_H_34_O_4_	3.56	2.75E-04	22.15	↑	—	31679	—
100	LysoPC(20:5 (5Z,8Z,11Z,14Z,17Z))	10.64	541.6570	C_28_H_48_NO_7_P	1.73	1.88E-02	1.65	↑	—	10397	+
101	15(S)-HPETE	10.49	336.4657	C_20_H_32_O_4_	3.91	1.22E-04	4.49	↓	—	4,244	—
102	Estrone	10.26	270.3661	C_18_H_22_O_2_	4.97	1.08E-05	4.93	↓	—	145	—
103	11,12,15-THETA	10.18	354.4810	C_20_H_34_O_5_	4.67	2.15E-05	3.48	↑	—	4,684	—
104	Palmitic acid	9.55	256.4241	C_16_H_32_O_2_	2.83	1.47E-03	1.29	↑	—	220	+
105	11b-Hydroxyprogesterone	9.46	330.4611	C_20_H_28_O_4_	4.70	2.00E-05	6.23	↓	—	4,031	—
106	Androstenedione	9.31	286.4085	C_19_H_26_O_2_	1.73	1.87E-02	5.19	↓	—	53	—
107	Allopregnanolone	9.16	363.2590	C_21_H_34_O_2_	1.42	3.77E-02	7.00	—	↓	1,449	—
108	Pyridoxamine 5′-phosphate	0.80	249.0656	C_8_H_13_N_2_O_5_P	1.87	1.33E-02	1.17	—	↑	1,555	+
109	Paraxanthine	0.78	180.1640	C_7_H_8_N_4_O_2_	2.96	1.09E-03	1.53	↓	↑	1860	+
110	Indoleacetic acid	0.75	176.0683	C_10_H_9_NO_2_	2.14	7.17E-03	1.14	—	↑	197	+
111	Pyridoxamine	0.70	168.1931	C_8_H_12_N_2_O_2_	2.17	6.73E-03	1.52	↓	—	1,431	—
112	15-KETE	0.17	341.2050	C_20_H_30_O_3_	2.81	1.55E-03	1.51	—	↑	10210	+
113	DG (18:2 (9Z,12Z)/20:1 (11Z)/0:0)	0.05	647.5553	C_41_H_74_O_5_	1.41	3.87E-02	1.54	—	↓	7,253	+

**FIGURE 7:**
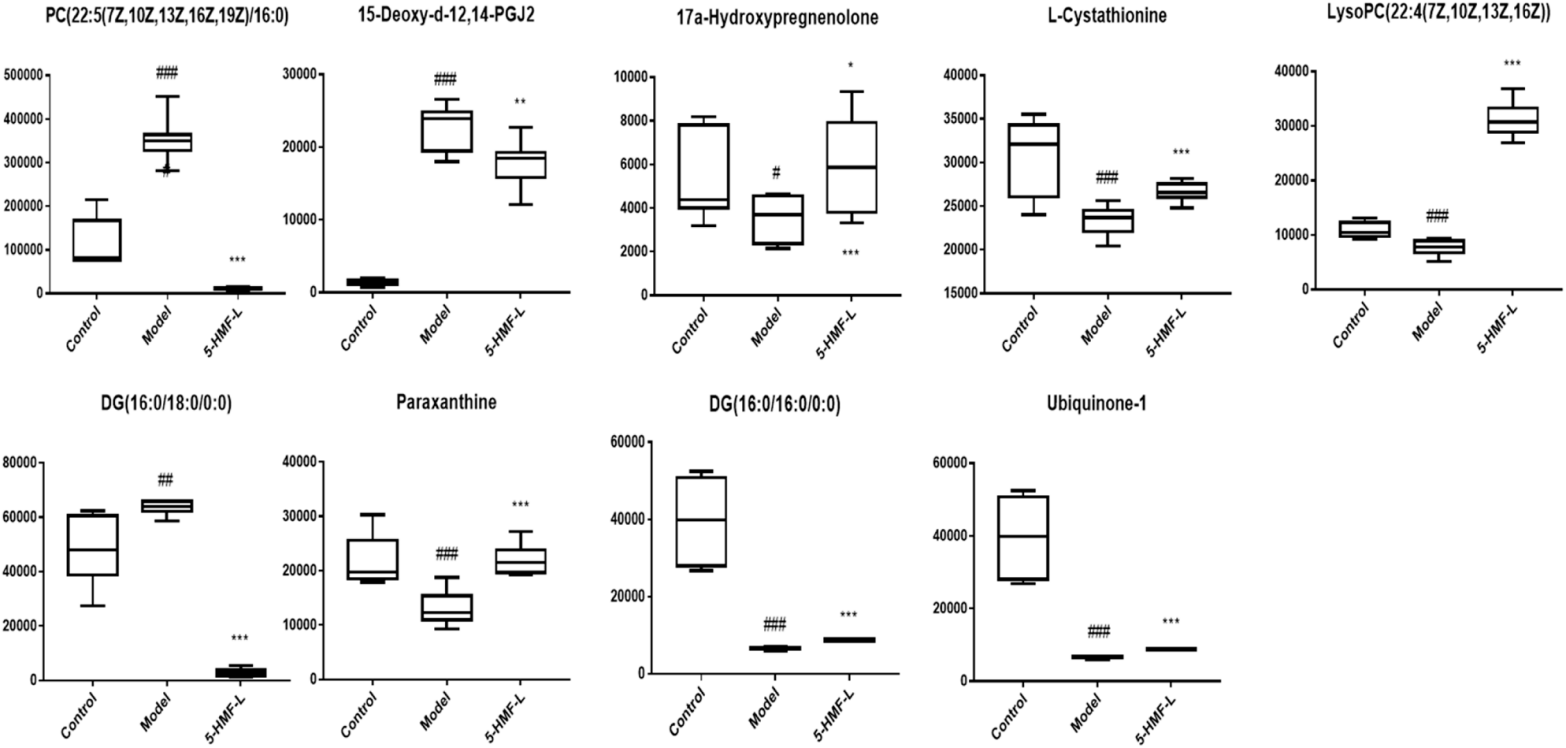
Comparison of the relative content of different metabolites in plasma of the different administration groups. All data are expressed as mean ± standard deviation (*n* = 8). ^*#*^ *p* < 0.05, ^*##*^ *p* < 0.01, ^*###*^ *p* < 0.001 compared with the control group; *p < 0.05, ***p < 0.001 compared with the model group.

**FIGURE 8:**
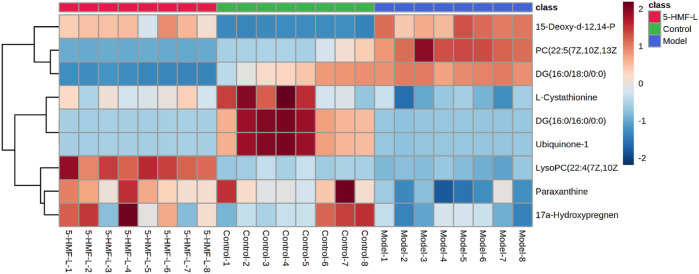
Cluster analysis heatmap of different metabolites in BDS rat plasma. Brown and blue represent higher and lower than average levels of different metabolites, respectively. The rows represent different metabolites, and the columns represent rat plasma samples (*n* = 8).

There were 9 metabolites involved in metabolism in the control group, model group, and 5-HMF-L group (Figure 7). To further clarify the distribution of 9 different metabolites in different groups, unsupervised clustering was performed by using a hierarchical clustering analysis heat map. As shown in Figure 8, there were significant differences between the model group and the control group. These metabolites could be significantly regulated, and metabolic disorders in rats after 5-HMF treatment were improved.

#### 3.5.3 Analysis of Metabolic Pathways

**FIGURE 9:**
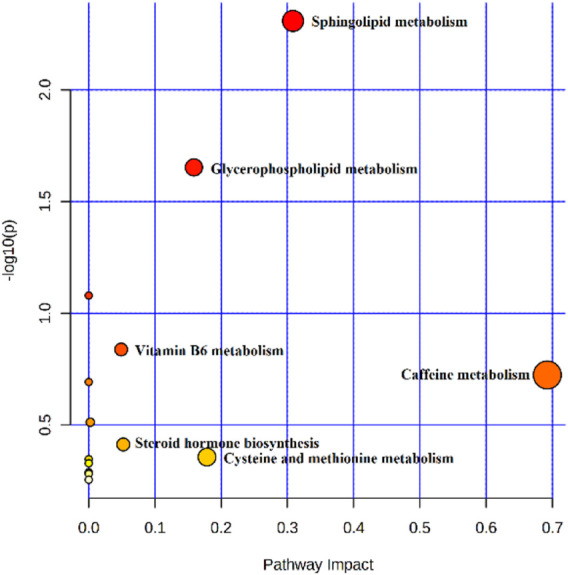
Pathway analysis of plasma samples from BDS rats.

The metabolites of the 5-HMF-L group were input into MetaboAnalyst to explore the metabolic pathways of 5-HMF in the treatment of BDS. The results showed that the 5-HMF-L group may play a role through metabolism of cysteine, methionine, glycerophospholipid, sphingolipid, caffeine, vitamin B6, glycerol, and tyrosine and steroid hormone biosynthesis (Figure 9).

#### 3.5.4 Pathway Analysis of Targets and Metabolites

**FIGURE 10:**
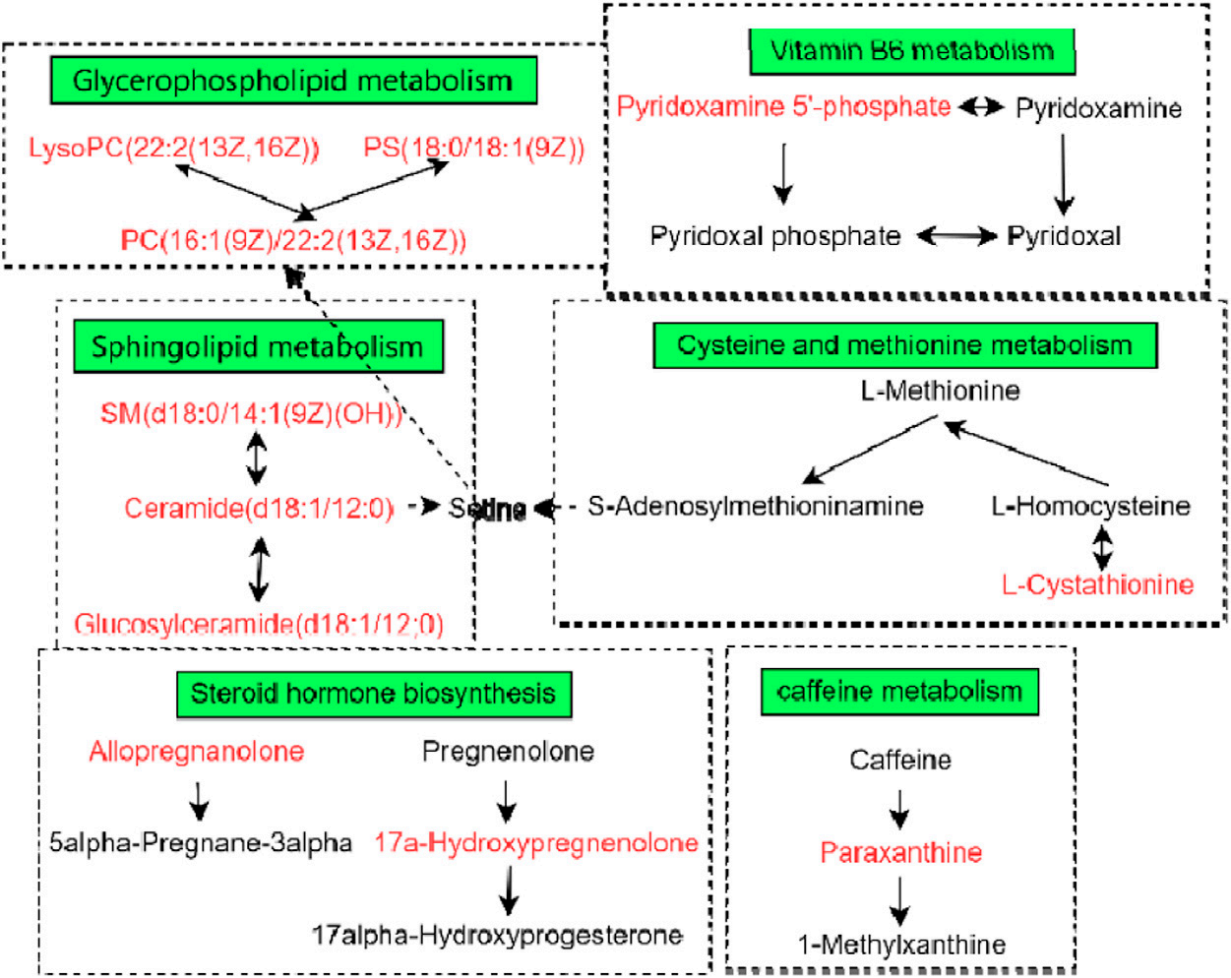
Pathway analysis of plasma samples in 5-HMF-L group.

To explore the crucial metabolic pathways, joint pathway analysis by MetaboAnalyst was conducted with the 57 differential metabolites and 84 targets. The results showed that only steroid hormone biosynthesis was simultaneously enriched with the targets from network pharmacology and the differential metabolites from metabolomics. According to the number of targets from the network pharmacology in the pathway, steroid hormones biosynthesis was selected as the most crucial metabolic pathway (Figure 10).

### 3.6 Effects of 5-Hydromethylfurfural on the Expression of Key Proteins in the Spleen of Blood Deficiency Rats

**FIGURE 11:**
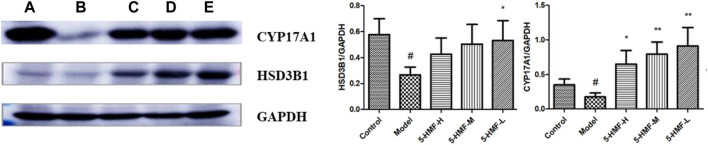
Effect of each group on protein expression. **(A)** Control group, **(B)** Model group, **(C)** 5-HMF-H group, **(D)** 5-HMF-M group, **(E)** 5-HMF-L group. ^*#*^ *p* < 0.05, compared with the control group; *p < 0.05 and **p < 0.01, compared with the model group.

Compared with the control group, the expression of CYP17A1 and HSD3B1 protein in the spleen of the model group was decreased (*p* < 0.05). Compared with the model group, the 5-HMF-H group had increased expression of CYP17A1 protein (*p* < 0.05), and the 5-HMF-M and 5-HMF-L groups had significantly increased expression of CYP17A1 protein (*p* < 0.01). Compared with the model group, the 5-HMF-L group had increased expression of HSD3B1 protein (*p* < 0.05) (Figure 11).

## 4 Discussion

According to the theory of TCM, the pathogenesis of BDS might be qi-blood discord, the imbalance between yin and yang, blood stasis, and visceral dysfunction. Therefore, Chinese herbal medicines that could nourish yin and blood, fill the essence, and nourish the marrow were often used clinically to treat BDS and anemia (Bailly et al., 1978). Another medicine, RP, has been used for more than 1,000 years to nourish yin and blood, but the possible mechanism of the effect of “tonifying blood” is still unclear. The appearance of rats in the model group changed significantly, including hair loss, slow movement, and weight loss. From the behavior of BDS rats, 5-HMF was effective in treating BDS. Peripheral RBC, WBC, HGB, and HCT were significantly reduced, a typical clinical manifestation of BDS and anemia (Safeukui et al., 2015). At the same time, the 5-HMF-H group did not have increases in WBC, RBC, and HGB, but the 5-HMF-M group and the 5-HMF-L group had more obvious therapeutic effects. Another indication of anemia is splenomegaly, which is one of the most common and earliest pathological manifestations in different types of anemia (Altamura et al., 2019). It was generally believed that hematopoietic dysfunction was related to the discharge of bone marrow cells from bone marrow through blood, resulting in accumulation in the spleen (Koldkjaer et al., 2013). The collection of RBCs in the enlarged spleen could exacerbate anemia. Therefore, the reduction of RBCs in circulation and anemia are related to the degree of spleen enlargement (Gilep et al., 2011). According to Figure 2, the spleen mass in the model group was significantly greater than that in the control group. At the same time, compared with the model group, the spleen of the rats treated with 5-HMF was not enlarged. This showed that 5-HMF could reduce the side effects of chemotherapy on the spleen.

First, using the network pharmacology method, the therapeutic effect of 5-HMF on BDS was analyzed from the two perspectives of target and pathway. From the results of network pharmacology, the regulation of metabolic pathways might be one of the main mechanisms in 5-HMF treatment of BDS. Second, from the perspective of metabolomics, 5-HMF did have a curative effect on BDS, and the curative effect in the 5-HMF-L group was greater than that in the other two groups. Therefore, it showed the efficacy of 5-HMF in BDS treatment, and the most effective dose was in the 5-HMF-L group. Combined with network pharmacology and metabolomics analysis, the steroid hormone biosynthesis may be the key pathway of 5-HMF in the treatment of BDS.

Steroids are commonly used drugs to treat autoimmune hemolytic anemia, but long-term use of steroids can lead to drug resistance (Schiffer et al., 2019). Steroid hormones play a vital role in regulating water and salt balance, metabolism and stress response, and initiating and maintaining sexual differentiation and reproduction (Porcu et al., 2016). The production of new steroids produces steroid hormones in the adrenal cortex, gonads, and placenta. In addition, a series of neurosteroids are produced in the brain (Christakoudi et al., 2018; Sackett et al., 2018). They can promote the body’s hematopoietic function, increase HGB, accelerate the circulation of oxygen and CO_2_, and enhance physical strength (Wu et al., 2016).

The metabolic pathway of steroid hormone biosynthesis was entered into the KEGG database and compared with the critical metabolic pathway genes predicted by network pharmacology. We found that CYP17A1 and HSD3B1 are essential proteins for steroid synthesis (Almassi et al., 2018). Western blot verified that in the 5-HMF-L group, the expression of CYP17A1 and HSD3B1 proteins was significantly up-regulated, which was consistent with the changing trends of metabolic results, verifying v the reliability of the metabolomics and network pharmacology prediction. Therefore, we have reason to believe that the steroid hormone biosynthesis pathway may be related to BDS and may be the potential mechanism of 5-HMF in the treatment of BDS. Our results suggest that 5-HMF provides a new therapeutic target by regulating related signaling pathways.

## 5 Conclusion

Our data showed that 5-HMF could ameliorate blood deficiency in rats induced by chemotherapy. Its mechanism might be regulating the expression of CYP17A1 and HSD3B1 in the steroid hormone biosynthesis pathway. These findings suggest that 5-HMF may be an effective alternative drug for BDS treatment.

## Data Availability

The raw data supporting the conclusions of this article will be made available by the authors, without undue reservation.

## Abbreviations

SD, Standard deviation; EDTA, Ethylenediaminetetraacetic acid; TCMSP, Traditional Chinese Medicine Systems Pharmacology Database and Analysis Platform; OMIM, Online Mendelian Inheritance in Man; KEGG, Kyoto Encyclopedia of Genes and Genomes; GO, Gene Ontology; VIP, Variable importance in the projection.
